# Supplementary material for: Facilitating stakeholder engagement in early stage translational research
Source: PLoS One. 2020 Jul 2;15(7):e0235400. doi: 10.1371/journal.pone.0235400 (PMC7332000; doi:10.1371/journal.pone.0235400)
Supplement: S1 Table — (DOCX) [file pone.0235400.s001.docx]

| Applications or uses of work | | | |
| --- | --- | --- | --- |
|  | Clinical/bedside application | | |
|  | NGO/Non-profit | | |
|  | Other | | |
|  | Public policy | | |
|  | T1/T2 research: guide, build, inform | | |
|  | T3/T4 research: guide, build, inform | | |
| Decisions made in work | | | |
|  | Outcomes of decisions on work | | |
|  | Types of decisions | | |
|  | Who makes decisions | | |
| End users affected by work | | | |
|  | Citizens/Public | | |
|  | | | Patients with chronic or acute illness |
|  | Industry | | |
|  | NGOs | | |
|  | Payers | | |
|  | PIs T1/T2 | | |
|  | PIs T3/T4 | | |
|  | Policymakers | | |
|  | Providers | | |
|  | Purchasers | | |
| Recommendations, wishes | | | |
| Sample Description | | | |
|  | Description of Research | | |
|  | Title/Field | | |
| Work Context | | | |
|  | Challenges/barriers to work | | |
|  | Collaborations: providers, res, orgs | | |
|  | Facilitators to work | | |
|  | Funding | | |
|  | Government initiative | | |
|  | Institutional support | | |
|  | | CTSI/CTSA | |
|  | Real-world application/Pub H problem | | |

Table 1. Codebook
