# Supplementary material for: Facilitating stakeholder engagement in early stage translational research
Source: PLoS One. 2020 Jul 2;15(7):e0235400. doi: 10.1371/journal.pone.0235400 (PMC7332000; doi:10.1371/journal.pone.0235400)
Supplement: S1 Appendix — (DOCX) [file pone.0235400.s002.docx]

Appendix A. Discussion Guide

**Focus Group Discussion Guide**

**Title: Developing a Framework for Stakeholder Engagement in early stage Translational Research**

**Time: 1 hr**

**Attendees: 1 Facilitator, 1-2 Additional Staff, 3-6 participants**

**5 minutes Prior to Start (Slide 1)**

Begin the meeting on WebEx at least 5 minutes prior to the start time and share the screen with the title slide visible (Slide 1).

Verify audio and visual are working.

# Introduction and Explanation (10 minutes)

## Welcome

Thank you for your interest and willingness to participate in this discussion.

My name is Amy LeClair, and I will be a co-facilitator of the discussion today. I have been at Tufts Medical Center since the Spring of 2015. I am a Senior Research Associate in the Institute for Clinical Research and Health Policy Studies, where my focus is on health services research. My background is as a medical sociologist with experience in qualitative and mixed methods research, and I also work with Tufts CTSI on a number of projects related to Stakeholder and Community Engagement. I am very excited to be here today to hear your thoughts and get your input on these topics.

**Agenda**

*Begin slide deck and advance to “Agenda” slide.*

On the screen is our agenda.

By way of introductions, I want to tell you first about who we are and what we hope to accomplish today. I will then ask the rest of the team to introduce themselves.

Next, we’ll spend a few minutes on establishing ground rules and your consent to participate in this discussion.

We will then launch into the discussion. This discussion will follow specific, pre-planned questions. We are keeping time and may decide to move through some questions quickly in order to address every topic on our list.

**Introduction**

*Advance to* selected *“Introductions” slide*

Tufts Clinical and Translational Sciences Institute is one of 6) “hubs” across the country funded by the National Center for Advancing Translation Sciences, or NCATS. Stakeholder and Community Engagement is one of our “signature programs” here at the Tufts CTSI. You have been invited to take part in this focus group because we are interested in learning more about Stakeholder and Community Engagement in early stage translational science.

*Ask Co-Facilitator to introduce themselves*

I am a Senior Policy Researcher at the RAND Corporation and Assistant Professor at Tufts University School of Medicine, and I am also the director of Stakeholder and Community Engagement in the Tufts’ CTSI. In my research I focus on improving health care for patients with high-cost clinical conditions and on involving patients and other stakeholders in research

Now we’d like to ask each of you to introduce yourselves by name, and if you wish, title and institutional affiliation, and maybe tell us a little bit about your research.

Now we’d like to ask each of you to introduce yourselves by name, organization, and a little about the type of research you do.

**Ground rules and consent**

I'd like to talk about a few ground rules and make sure that everyone consents to participate in the discussion.

*Advance to “Ground Rules” slide.*

In this discussion, we will ask you to speak about your own views, informed by your experience as a researcher. In a few cases, we may ask how you think your peers – other researchers and scientists – might view something. In all other cases, please assume our questions ask *you* to describe *your own* experience and views. Finally, it would be helpful if you would state your *first* name each time you speak.

*Advance to “Consent” slide.*

Prior to joining us today, everyone should have received an “Information Sheet” detailing what we are asking of you and your rights to confidentiality. We also have copies available today if anyone would like one. This study has been reviewed by Tufts Medical Center’s IRB and deemed exempt.

Tufts CTSI will use the information you provide during this focus group for research purposes only. Everything you say in this discussion will be treated confidentially. In our reports, comments will not be attributed to specific individuals or organizations. In conversations you have outside of the focus group, we request that you also protect the confidentiality of others in the group. Please do not repeat anything that is said here in a way that is attributable to a particular person or organization. As you decide what to share with others during the discussion, however, keep in mind that Tufts CTSI cannot guarantee that everything you say will be kept confidential by all of the participants.

And finally, we're audio recording the session because we don't want to miss any of your comments. The recordings will be used to create de-identified transcripts; after the transcripts have been created, the recordings will be destroyed. The de-identified transcripts will be the basis for our analysis. The names of individuals who participated in these focus groups will also remain confidential.

Does anyone have any questions?

Now I will ask each one of you in turn if you consent to audio recording. Please answer yes or no.

XX, do you consent to audio-recording of the discussion today?

*If yes, record beginning time.*

# Decision Making (20 minutes)

Our first topic of discussion is decision making. I’d like to start by asking about the decision making process in your research:

1. What decisions and questions is your research designed to inform?

*Probes*:

2. Who are the decision makers responsible for these decisions?

*Probes*:

3. What information is needed to make those decisions?

*Probes:*

4. What groups and/or individuals are affected by these decisions?

*Probes:*

# Scientific Discovery (20 minutes)

Now we’d like to talk about scientific discovery. As researchers, you are in the business of scientific discovery.

5. Who or what is most influential in setting the agenda for priorities in scientific discovery?

*Probes*

6. Who or what do you hope to inform with your findings?

*Probes*

- 1. Can you think of specific examples?

# Conclusion (5 minutes)

Discussion recap

Final thoughts or questions

*Advance to “Thank you for Participating” slide*

Thank you, everyone for taking time to participate in today’s focus group. Your feedback has been incredibly informative and we appreciate your time and efforts. If you have any questions, please contact Amy LeClair at [aleclair@tuftsmedicalcenter.org](mailto:aleclair@tuftsmedicalcenter.org)
