# Supplementary material for: Facilitating stakeholder engagement in early stage translational research
Source: PLoS One. 2020 Jul 2;15(7):e0235400. doi: 10.1371/journal.pone.0235400 (PMC7332000; doi:10.1371/journal.pone.0235400)
Supplement: Attachment — Submitted filename: RESPONSE TO REVIEWERS.docx [file pone.0235400.s003.docx]

RESPONSE TO REVIEWERS

Dear Karen-Leigh Edward and reviewers,

Thank you for the thoughtful reviews and opportunity to respond. We have organized our responses below:

1. **Style requirements** – We have reviewed our files to ensure they meet PLOS ONE’s style requirements.
2. **Data availability** – While we understand data sharing has become more common for qualitative data, we are new to the concept for the sharing of qualitative data. We realize, in retrospect, we should have addressed this when submitting the manuscript, and the lead author – Dr. Amy LeClair – consulted with the Qualitative Data Repository (QDR) group for guidance. What we should have said in the original submission is that the data cannot be shared. We appreciate the opportunity to learn from this experience, both for the submission of future manuscripts and for the design of qualitative studies going forward.
   a. This study was deemed exempt by Tufts Health Sciences Institutional Review Board, therefore a written informed consent was not used. However, participants were given an information sheet and verbal consent was obtained prior to beginning the focus groups/interviews. During this process, participants were explicitly told that their data would not be shared outside of the research team, and that only de-identified excerpts of the transcripts would be used in publication and other forms of dissemination. Furthermore, the complete data set from all four sites was only available to three members of the team for coding.
3. **ORCID ID** – The corresponding author now has an ORCID ID, and it has been validated in Editorial Manager.
4. **Competing Interests** – The RAND Corporation is not a commercial company. RAND has the same nonprofit status as the academic institutions and medical centers the other authors’ are affiliated with. It would not be appropriate to declare this as a competing interest and is not the policy of RAND or its researchers and staff. We have, therefore, not updated any of our Funding Statements or Competing Interests Statements
5. **Supporting Information** – We have including captions for our supporting Information files.
6. **Reviewers**’ **Comments**:

| Comment | Response |
| --- | --- |
| Academic Editor: “Please made overt the framework used to inform the maintenance of rigour throughout the qualitative data collection and analysis. Also further detail about how the themes emerged is required.” | We have made several edits to the 3^rd^ paragraph of the methods section to clarify the framework that guided our data collection and analysis, including adding citations to the literature that guided our methods. We hope this clarifies the methodology. |
| Reviewer #1: The terms CTSA, T0, T1, T2, should be explained the first time each is used in the manuscript. | We have made sure all acronyms and abbreviations are spelled out in their first use and have also added a citation for our definitions of T0 and T1 in the 2^nd^ paragraph of the article. |
| On page 2 where it says 'are three actors can help" should read "are three factors". | We have changed the word “actors” to “organizational entities” to more clarify t our meaning. |
| On page 11 where it says "by the set of pants" should probably read "by seat of pants" | Yes – thank you for picking up that type. We have made the correction. |
| On page 14 "we describe the actors who may take charge". Actors should be changed to a more scientific term such as participants or entities. | We have changed “actors” to “organizations.” |
| I thought the parts "Addressing definitions" and "Instruction manual" were both accurate and insightful. The section "Focusing on CTSA hubs...research", clearly outlines hoe limited access to funding affects all aspects of the scientific research process. | Thank you. |
| Reviewer #2: The major concern about the current study is qualitative and the findings were from interviews and subjective. The topic is interesting, if conduct the study by design a survey with questionnaire and collect the data from the identified stakeholders, and then analyze the data, according to the response rate, the reliability and validity would allow the study more informative and objective. | Per the Academic Editor – “Please disregard the reviewer 2 comment about quantitative research as this does not apply to your paper.” |
